# MicroRNA-122 Modulates the Rhythmic Expression Profile of the Circadian Deadenylase *Nocturnin* in Mouse Liver

**DOI:** 10.1371/journal.pone.0011264

**Published:** 2010-06-22

**Authors:** Shihoko Kojima, David Gatfield, Christine C. Esau, Carla B. Green

**Affiliations:** 1 Department of Neuroscience, University of Texas Southwestern Medical Center, Dallas, Texas, United States of America; 2 Department of Biology, University of Virginia, Charlottesville, Virginia, United States of America; 3 Department of Molecular Biology, University of Geneva, Geneva, Switzerland; 4 Regulus Therapeutics, Carlsbad, California, United States of America; Vanderbilt University, United States of America

## Abstract

Nocturnin is a circadian clock-regulated deadenylase thought to control mRNA expression post-transcriptionally through poly(A) tail removal. The expression of Nocturnin is robustly rhythmic in liver at both the mRNA and protein levels, and mice lacking Nocturnin are resistant to diet-induced obesity and hepatic steatosis. Here we report that Nocturnin expression is regulated by microRNA-122 (miR-122), a liver specific miRNA. We found that the 3′-untranslated region (3′-UTR) of *Nocturnin* mRNA harbors one putative recognition site for miR-122, and this site is conserved among mammals. Using a luciferase reporter construct with wild-type or mutant *Nocturnin* 3′-UTR sequence, we demonstrated that overexpression of miR-122 can down-regulate luciferase activity levels and that this effect is dependent on the presence of the putative miR-122 recognition site. Additionally, the use of an antisense oligonucleotide to knock down miR-122 *in vivo* resulted in significant up-regulation of both *Nocturnin* mRNA and protein expression in mouse liver during the night, resulting in Nocturnin rhythms with increased amplitude. Together, these data demonstrate that the normal rhythmic profile of *Nocturnin* expression in liver is shaped in part by miR-122. Previous studies have implicated Nocturnin and miR-122 as important post-transcriptional regulators of both lipid metabolism and circadian clock controlled gene expression in the liver. Therefore, the demonstration that miR-122 plays a role in regulating *Nocturnin* expression suggests that this may be an important intersection between hepatic metabolic and circadian control.

## Introduction

Nocturnin is a circadian deadenylase [1], [2], which removes poly(A) tails from its target RNAs, and is thought to control target RNA expression by either enhancing RNA degradation or silencing translation. *Nocturnin* shows rhythmic expression in many tissues such as spleen, kidney and heart in mice and this rhythmicity is particularly robust in liver [3]. *Nocturnin* is also an immediate early gene, and its expression is acutely induced by stimuli such as serum and 12-O-tetradecanoyl-phorbol-13-acetate (TPA) in cultured cells [2]. Mice lacking *Nocturnin* (*Noc* ^−/−^) are resistant to diet-induced obesity and hepatic steatosis [4]. Although the expression of ‘core’ circadian clock genes is not affected in *Noc* ^−/−^ mice, expression profiles of rhythmic ‘output’ genes such as *Srebp1c* and *Pparγ*, key regulators of lipid-related gene expression, are significantly changed [4]. These and other data indicate that Nocturnin is important for proper lipid metabolism.

MicroRNAs (miRNAs) are short (19–25 nt), noncoding RNA molecules that can regulate their target gene expression post-transcriptionally [5]. MiRNAs are transcribed, capped, adenylated and spliced just as protein-coding mRNAs, and these primary transcribed miRNAs (pri-miRNAs) are then cleaved by *Drosha*, which is an RNaseIII endonuclease, in the nucleus, releasing the shorter (−65 nt long) precursor miRNA (pre-miRNA). Subsequently, pre-miRNAs are exported into the cytoplasm, and are further digested by *Dicer*, which is an endonuclease that cleaves double-strand RNA or pre-miRNAs, and become a short duplex RNA. This duplex RNA is incorporated into the functional miRNA-induced silencing complex (miRISC) where target mRNAs are recognized and processed [6], [7]. miRNAs recognize target sequences usually in the 3′-UTRs of target mRNAs with a requirement for a nearly perfect match between the 5′-proximal ‘seed’ region (position 2–8) of the miRNA and its target mRNA for binding specificity [7], [8]. Once the miRNA recognizes and interacts with its target RNA, it generally results in inhibition of protein synthesis and/or triggers deadenylation and degradation, although the detailed mechanisms are still in debate [6], [7].

In mammals, more than 50% of all protein-coding mRNAs are predicted to be targets of a miRNA, therefore most, if not all, biological processes seem to be controlled by miRNAs to some degree [6]. Contributions of circadian clock function to miRNA or *vice versa* have been demonstrated. Rhythmic expression of miRNAs has been observed in mouse retina and suprachiasmatic nucleus (SCN), the site of the circadian pacemaker [9], [10]. Other miRNAs such as miR-132 and miR-219 are functionally involved in the clock and regulate circadian period or light dependent resetting of the clock in the SCN [9]. Also several mammalian core clock genes such as *Period1*, *2*, *3* and *Clock* as well as *Drosophila Clock* have been demonstrated to be targets of miRNAs [11], [12], [13].

MicroRNA-122 (miR-122) is a liver-specific miRNA. It is the most abundant miRNA in this organ accounting for approximately 70% of the total miRNA population [14]. Proper miR-122 expression is important for normal liver function [15], [16]. Blocking miR-122 expression leads to the reduction of plasma cholesterol and triglyceride levels in both rodents and primates, [15], [17], [18], [19]. Moreover, mice in which miR-122 is knocked down are resistant to diet-induced hepatic steatosis [18], supporting a role for this miRNA in fatty acid and cholesterol metabolism in liver. In humans, the level of miR-122 is repressed in hepatocellular carcinomas and in patients with nonalcoholic steatohepatitis [20], [21]. There is also a functional connection between miR-122 and circadian rhythms, because the transcription of miR-122 is rhythmic, and circadian transcripts are enriched in a gene set that is misregulated by miR-122 knock-down *in vivo* [22].

Many mRNAs have been predicted to be potential targets of miR-122, but only a few genes such as cationic amino acid transporter1 (Cat1), cyclinG1, or Smarcd1/Baf60a have been demonstrated to be *bona fide* targets [15], [22], [23], [24], [25]. In this study, we identified *Nocturnin* as one of the target mRNAs of miR-122 in mouse liver and we propose that miR-122 is important for shaping the appropriate circadian expression profile of *Nocturnin*.

## Results and Discussion

### *Nocturnin* is a target of miR-122 in cultured cells

**Figure 1:**
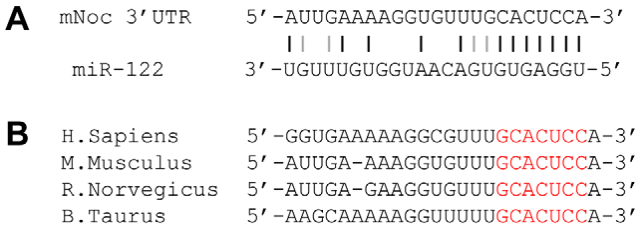
The *Nocturnin* 3′UTR possesses one putative miR-122 recognition site. **A**. The sequence of WT *Nocturnin* 3′-UTR (top) and miR-122 (bottom) around the putative miR-122 recognition site. Black and gray lines represent the perfect match and G-U wobbles, respectively. **B**. miR-122 recognition sequences of *Nocturnin* gene in Homo sapiens (NM_012118; nt1930–1953), Mus musculus (NM_009834, nt2100–2121), Rattus norvegicus (NM_138526, nt1680–1702), and Bos taurus (NM_001082454, nt1693–1715). Red characters represent the seed sequence for miR-122 recognition.

Although target mRNAs frequently have multiple copies of miRNA target sites in their 3′-UTR [7], examination of the *Nocturnin* mRNA sequence revealed a single potential target sequence for miR-122 in its 3′-UTR (Fig. 1A). However, this single site contained a miR-122 seed sequence that was highly conserved in human, mouse, rat, and cow (Fig. 1B).

**Figure 2:**
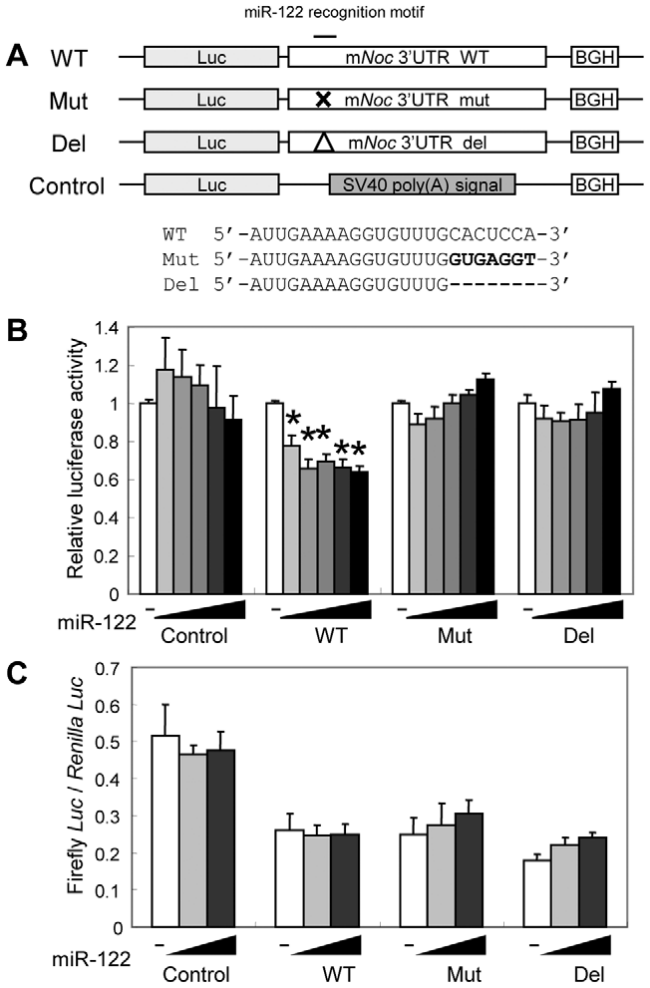
miR-122 down-regulates *Nocturnin* WT luciferase reporter activity but not its RNA level. **A**. Schematic representation of the *Nocturnin* 3′-UTR luciferase reporter genes. Nucleotide sequences of mutations introduced into Mut and Del reporters are also shown. X and Δ denote mutation and deletion of WT *Nocturnin* 3′-UTR, respectively. Luc; luciferase, BGH; Bovine growth hormone polyadenylation signal. **B**. Relative luciferase activities (means ± S.E. Three independent experiments in duplicate) of *Nocturnin* 3′-UTR reporters with various levels of miR-122 overexpression in NIH3T3 cells. *Firefly* luciferase activity was normalized to *Renilla* luciferase activity. The *firefly*/*Renilla* ratios without miR-122 expression were set as 1 for each reporter gene. Asterisks represent p<0.005 versus no miR-122 overexpression (white bars). **C**. Relative RNA levels of reporter genes (means ± S.E. Two independent experiments in duplicate) with miR-122 overexpression in NIH3T3 cells. *Firefly luciferase* level was normalized to *Renilla luciferase* level.

In order to test whether this sequence was indeed a miR-122 recognition site, we used a cell-based luciferase reporter system in which the mouse *Nocturnin* 3′-UTR was cloned downstream of the *Firefly* luciferase gene. We also generated constructs in which the 7 bp “seed” sequence (CACUCCA) within the putative miR-122 site was either mutated or deleted (Fig. 2A). When miR-122 was expressed in NIH3T3 cells with the WT *Nocturnin* 3′-UTR, we observed a dose-dependent decrease in relative luciferase activity (Fig. 2B). In contrast, miR-122 overexpression did not affect the luciferase activity of constructs with mutated or deleted miR-122 target sequences (Fig. 2B). Similar results were obtained in HEK293 cells as well (data not shown). These results indicated that *Nocturnin* is a potential direct target of miR-122, and miR-122 recognizes the putative recognition site present in the *Nocturnin* 3′-UTR.

Since most mRNAs undergo either deadenylation and degradation and/or inhibition of protein synthesis after miRNA recognition and binding [6], [7], we investigated whether miR-122 overexpression affected the *Nocturnin* 3′-UTR reporter RNA levels. Although the overall RNA levels of control reporter (lacking any of the *Nocturnin* 3′-UTR) were higher than those containing the 3′-UTR sequence, in no case was there an effect of miR-122 (Fig. 2C). Therefore, the changes in luciferase activity must be due to miR-122 mediated inhibition of protein synthesis rather than to RNA decay.

### Interaction between *Nocturnin* and miR-122 is specific

**Figure 3:**
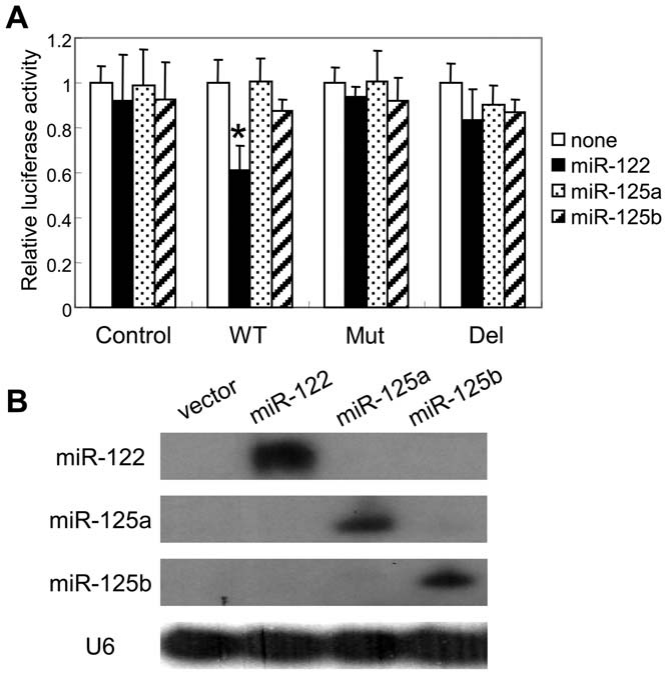
Effect of miR-122 on *Nocturnin* reporter expression is specific. **A**. Relative luciferase activities (means ± S.E. Two independent experiments in triplicate) when reporter genes were co-transfected with either miR-122, miR-125a, or miR-125b in NIH3T3 cells. Asterisks represent p<0.005 miR-122 versus no miR-122, miR-125a, or miR-125b. **B**. Northern blot analysis of miRNAs. Same amount of plasmid DNA (1 µg) to express miR-122, -125a, and -125b was transfected into HEK293 cells.

In order to investigate the specificity of miR-122 towards *Nocturnin*, we tested whether miR-125a or miR-125b could reduce the activity of the *Nocturnin* 3′-UTR reporter. These two miRNAs are highly expressed in brain, heart and lung, and some genes such as lin-28 and ERBBs have been identified as their targets [26], [27], [28], but the *Nocturnin* 3′-UTR does not contain putative miR-125a or 125b sequence recognition motifs. When these miRNAs were co-expressed with *Nocturnin* 3′-UTR reporter genes, both miR-125a and -125b failed to repress the luciferase activity of *Nocturnin* 3′UTR WT, even under conditions where miR-122 was able to effectively repress reporter gene expression (Fig. 3A). The failure to repress the *Nocturnin* 3′-UTR WT luciferase level by miR-125a or -125b was not due to the absence of miRNA expression, since Northern blot analysis showed that the miRNAs are well expressed in HEK293 cells (Fig. 3B).

### miR-122 expression is not rhythmic

**Figure 4:**
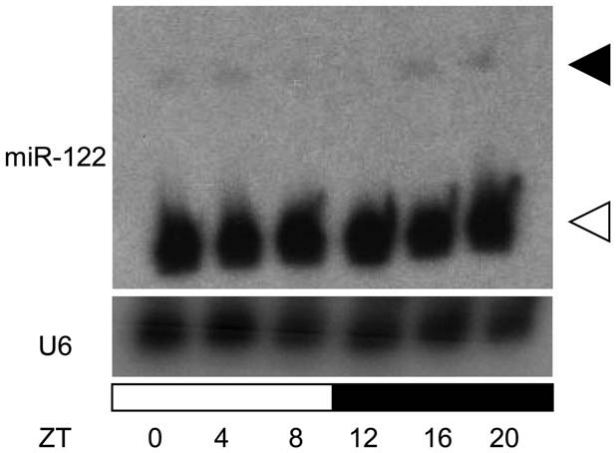
Mature miR-122 expression is not rhythmic in liver. The levels of miR-122 were determined by Northern blotting from liver samples taken at various circadian times as indicated. One mouse per time point was used. U6 snRNA was measured as a loading control. White and black arrowheads indicate mature miR-122 and pre-miR-122, respectively.

Since *Nocturnin* expression exhibits high amplitude rhythms in liver [3], [4], we tested whether miR-122 expression might also be rhythmic in liver. The mature form of miR-122 expression was not rhythmic (Fig. 4; white arrowhead), consistent with the fact that the half-life of miRNAs can be well over 24 hours [29]. However, we detected another more slowly migrating band in our Northern blot analysis, which was rhythmic with highest levels at night (Fig. 4; black arrowhead) and probably corresponded to pre-miR-122. These results are consistent with the previous report that the expression of both pri-miR-122 and pre-miR-122 is rhythmic but that mature miR-122 is not [22].

### The deadenylase activity of *Nocturnin* is not necessary for miR-122 mediated self regulation

**Figure 5:**
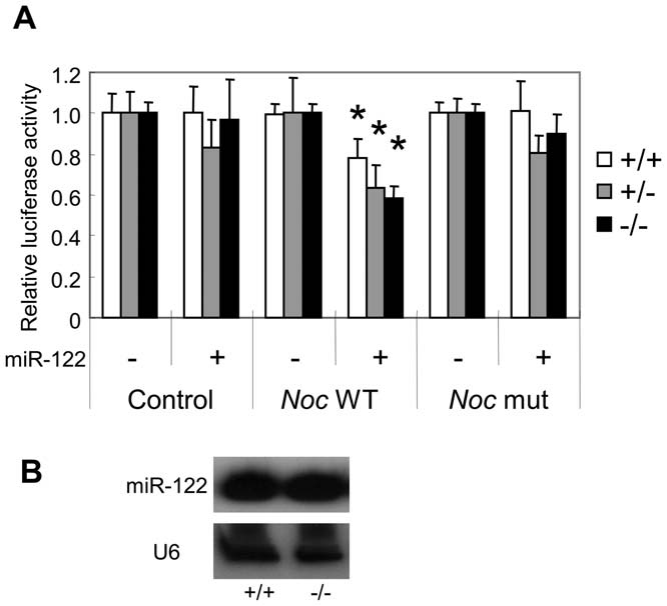
Deadenylase activity of *Nocturnin* was not involved in miR-122-mediated self-regulation. **A**. Relative luciferase activities (means ± S.E. Three independent experiments in duplicate) with miR-122 overexpression in MEFs (White bars; +/+, Gray bars; +/−, Black bars; −/−). Luciferase activities were normalized as described in Figure 2. Asterisks represent p<0.005 versus no miR-122. **B**. miR-122 expression was measured from *Noc* ^+/+^ and *Noc* ^−/−^ livers. RNA samples are pools of equal amounts of RNA from each circadian time point (ZT 0, 4, 8, 12, 16, and 20, one mouse per time point).

MiRNAs not only repress translation but can also trigger deadenylation and degradation and/or translational silencing of their target mRNAs [30], [31]. Since the *Nocturnin* gene encodes a deadenylase, we wondered whether Nocturnin's deadenylation activity could contribute to the miR-122 effect on the *Nocturnin* message. We thus co-expressed miR-122 and *Nocturnin* reporter genes (Fig. 2A) in Mouse Embryonic Fibroblasts (MEFs) derived from *Noc* ^+/+^, *Noc* ^+/−^, and *Noc* ^−/−^ mice to see if loss of a functional Nocturnin deadenylase would affect the down-regulation of *Nocturnin* expression mediated by miR-122. However, no significant difference in miR-122's ability to down-regulate the expression of *Nocturnin* 3′-UTR reporter was observed between genotypes (Fig. 5A), suggesting that the deadenylase activity of *Nocturnin* is not necessary for miR-122 to down-regulate *Nocturnin* expression. Loss of *Nocturnin* expression also did not affect the expression level of miR-122 in mouse liver (Fig. 5B).

### Endogenous *Nocturnin* is a target of miR-122 *in vivo*

To test whether the effects of miR-122 on Nocturnin in cell culture could also be observed *in vivo*, we analyzed endogenous expression of Nocturnin in liver using mice in which miR-122 expression was knocked down by injecting miR-122 specific antisense oligonucleotides (ASOs).

**Figure 6:**
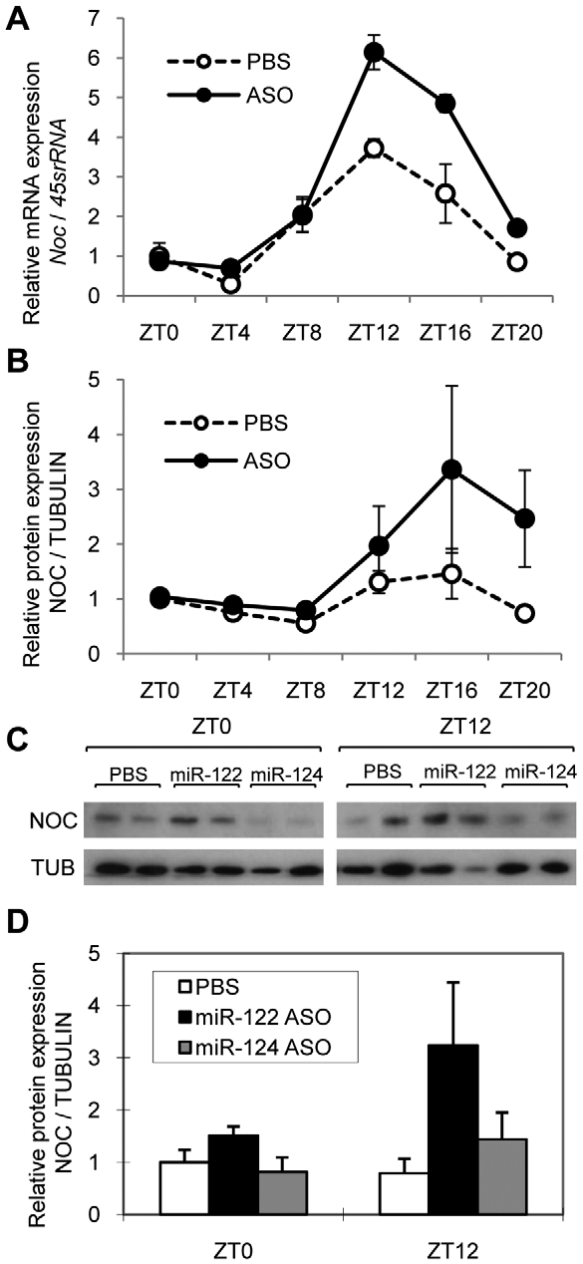
Nocturnin is up-regulated by miR-122 knock-down *in vivo*. **A**. *Nocturnin* mRNA expression was measured by qRT-PCR from livers collected at the times indicated from mice that were previously treated with miR-122 ASO or PBS (mean ± S.E.). Each time point represents an average from three mice. p<0.05 by two-way ANOVA (PBS vs. miR-122 ASO). **B**. Nocturnin protein expression in PBS or miR-122 ASO treated mouse liver (mean ± S.E.) around the clock was measured on Western blots and then quantitated using Image J software. Each point represents average of three mice. Two-way ANOVA (PBS vs. miR-122 ASO) was not significant (p = 0.32). **C**. Representative Western blots of Nocturnin expression in PBS, miR-122 ASO, or miR-124 ASO treated mouse liver (two mice per group) at ZT0 and ZT12. An independent set of mice as that in panels A and B was used. **D**. Nocturnin protein expression in PBS, miR-122 ASO, or miR-124 ASO treated mouse liver (mean ± S.E.) at ZT0 and ZT12. Western blots (Fig. 6C) were quantitated using Image J software. Each point represents an average of two mice.

Four doses of miR-122 ASO or PBS control were injected intraperitoneally into mice, and 14–15 days after the first injection (corresponding to 2–3 days after the last injection), livers were harvested around the clock at 4hr intervals. Analysis of livers from miR-122-depleted animals relied on the same samples as in [22]. As described in [22], miR-122 depletion was >85% on average, but showed some time point-dependent variation due to the rhythmic production of miR-122. *Nocturnin* mRNA expression remained rhythmic after miR-122 ASO administration, but the amplitude was significantly increased (Fig. 6A). This up-regulation was more significant at ZT12 and 16 (ZT refers to Zeitgeber Time and ZT0 is defined as time (hours) of lights on and ZT12 is defined as time of lights off) compared to other time points, corresponding to times when the expression of *Nocturnin* is usually high. These data were consistent with previous reports that listed *Nocturnin* mRNA as one of a set of mRNAs that were up-regulated by knocking down miR-122 *in vivo* [15], [18]. Although overexpression of miR-122 did not affect the RNA level of the *Nocturnin* reporter gene (Fig. 2C), the knock down of miR-122 was able to significantly increase *Nocturnin* mRNA levels in mouse liver. This could be due to the difference between synthetic reporter gene vs. endogenous mRNA or the difference between cultured cell system vs. *in vivo* system.

Endogenous Nocturnin protein expression was also measured around the clock with or without miR-122 ASO, and we found that Nocturnin protein was significantly higher during the night (ZT12) in the miR-122 ASO injected mice than in those injected with PBS (Fig. 6B). In an independent set of animals we furthermore confirmed that injection of a control ASO, specific for the brain-specific miR-124, did not increase Nocturnin levels at the two time points tested, ZT0 or ZT12 (Fig. 6C). The larger effect of miR-122 ASO at night is likely due to the high levels of *Nocturnin* mRNA during this phase of the circadian cycle. *Nocturnin* mRNA expression is tightly regulated in liver peaking at ZT12 (Fig. 6A), and its amplitude can be almost 100-fold [3]. Our data thus demonstrate that this robustly rhythmic expression of *Nocturnin* in liver is shaped, at least in part, by miR-122.

It has become increasingly clear that miRNAs play a role in many biological processes such as development, cell proliferation/differentiation, apoptosis, and metabolism, and misregulation of miRNA expression can lead to pathological conditions including cancer, autoimmunity, and obesity [6]. Obesity and/or metabolic syndrome have become a major health problem especially among western countries, and several reports have indicated that there is an association between miRNA expression and obesity and/or metabolic syndrome. For example, the expression of miR-27, -335, and -519d is up-regulated in liver and/or adipose tissue of obese mice or humans [32], [33], [34], [35], [36]. In addition, the proper expression of miR-122 in liver is critical for lipid metabolism, since overexpression of miR-122 increased and knockdown of miR-122 decreased cholesterol and fatty acid biosynthesis [15], [18]. Nocturnin is also important for proper lipid metabolism in liver, since *Noc* ^−/−^ mice manifest hepatic steatosis under high-fat diet conditions and are resistant to diet-induced obesity [4]. Furthermore, both miR-122 and *Nocturnin* are implicated to play a role in circadian rhythms [4], [22]. Taken together, our findings suggest that the regulation of Nocturnin expression by miR-122 is an important connection between circadian clocks and hepatic lipid metabolism.

## Materials and Methods

### Plasmid DNA constructs

The mouse *Nocturnin* 3′-UTR was cloned from mouse liver cDNA by PCR. The PCR was performed at 95°C for 5 min as the initial step and then for 30 cycles of 95°C for 30 s, 55°C for 30 s, and 72°C for 2 min, followed by a final extension of 1 cycle of 72°C for 7 min, utilizing the following primers; mNoc3UTRF 5′-CTAGATCACAAGCGTCTTAACCAGGG-3′, mNoc3UTRR 5′- TCTAGAACTTTATAAATAAGATATTTACCATTTTACCACTGG-3′. Then, the PCR product was cloned into pCR2.1-TOPO (Invitrogen). Subsequently, mutations into the putative miR-122 recognition site were introduced by QuikChange Site Directed Mutagenesis kit (Stratagene). The reactions were performed at 94°C for 5 min as the initial step and then for 18 cycles of 94°C for 30 s, 55°C for 1 min, and 68°C for 14 min, followed by a final extension of 1 cycle of 72°C for 7 min with the following primers; mNocmiR122mutF 5′- CCCGCATTGAAAAGGTGTTTGGTGAGGTGTTTGAGCTTGTTG-3′ and mNocmir122mutR 5′- CAACAAGCTCAAACTGGAGTGCAAACACCTTTTCAATGCGGG-3′ for m*Noc* 3′-UTR mut, and mNocmiR122delF 5′- CCCGCATTGAAAAGGTGTTTGGTTTGAGCTTGTTGTTCATCTGTG-3′ and mNoc miR122delR 5′- CACAGATGAACAACAAGCTCAAACCAAACACCTTTTCAATGCGGG-3′ for m*Noc* 3′-UTR del. All three *Nocturnin* 3′-UTR clones (WT, Mut, and Del) in pCR2.1-TOPO vector were verified by sequencing. Then, these inserts were removed from pCR2.1-TOPO by BamHI/ApaI digestion, and ligated into pELSB luciferase reporter vector [37].

For miRNA expression plasmids, genomic sequences of respective miRNAs were first amplified by PCR. The PCR was performed at 94°C for 5 min as the initial step and then for 40 cycles of 94°C for 30 s, 58°C (miR-122) or 55°C (miR-125a and miR-125b) for 30 s, and 72°C for 1 min, followed by a final extension of 1 cycle of 72°C for 7 min, utilizing the following primers; miR122F 5′-GTATGATGTGGTTTGTAAGAAGTGTCTGCC-3′, miR122R 5′-GTGTCAGGGTAGTCAGTGTTGGG-3′, miR125aF 5′-GCCAATGTCTCTAGGGTTCTAGAAGC-3′, miR-125aR 5′-CAGCTGGCAGACACGGAGGC-3′, miR125bF 5′-CCTGGGCCCACAGTAACAGTTG-3′, miR125bR 5′-GGGCCCCATTAACTGGCATATAATCC-3′. The PCR products were first cloned into pCR2.1-TOPO, and sequences were verified. Then, inserts were cut out by BamHI/EcoRV (miR-122 and miR-125a) or SpeI/NotI (miR-125b) and ligated into pcDNA3.1/V5-HisB (Invitrogen).

### Cells and Animals

Mouse Embryonic Fibroblast (MEF) cells were derived from E14 embryos of *Nocturnin* knockout mice (*Noc* ^−/−^), *Nocturnin* heterozygous mice (*Noc* ^+/−^) or their wild-type counterparts (*Noc* ^+/+^). All the MEFs, mouse NIH3T3 cells and human HEK293 cells were grown in Dulbecco's Modified Eagle Medium (Invitrogen) with 10% fetal bovine serum (ATLANTA biologicals) at 37°C with 5% CO_2_.

Mice were maintained on a 12∶12 LD cycles and fed *ad libitum*. Animal experiments were conducted following the protocols approved by the Institutional Animal Care and Use Committees. Antisense oligonucleotide (ASO) treatment was performed as previously described [22]. Briefly, ASO treatment was performed in 11-week male C57BL/6 mice (Elevage Janvier, Le Genest St Isle, France) by intraperitoneal injection. The ASOs were chimeric 2′-fluoro/2′-O-methoxyethyl modified oligonucleotides with a completely modified phosphorothioate backbone. The exact chemistry is available on request. Mice were allowed to adapt to a 12 hr/12 hr LD regimen for 10 days and then (days 11, 15, 18 and 22; at ZT6 or ZT22) received 4 doses of 20 mg ASO/kg body weight in 150 ml, or 150 ml of saline (PBS control). On days 24 and 25 (i.e. 2–3 days after the last injection), animals were sacrificed at the respective ZTs, and livers were snap-frozen in liquid nitrogen.

### DNA Transfections

Transfection was carried out using FuGENE6 (Roche) for NIH3T3 and HEK293 cells, or Lipofectamine 2000 for MEFs, according to manufacturer's instructions. Two days after transfection, cells were washed with phosphate-buffered saline (PBS) and harvested in 150 µl of passive lysis buffer (Promega). For induction of ecdysone-mediated transcription, Ponasterone A (5 µM) (Invitrogen) was added to the culture medium 20 h prior to harvesting the cells. Luciferase activities were assayed with the Dual-Luciferase Reporter Assay System (Promega) using Turner Designs Model 20 luminometer (Turner). The *Renilla* luciferase plasmid was cotransfected to normalize each transfection assay. For Fig. 2B and 3A, 24 well plates were used for luciferase measurement. Amounts of DNA that were transfected were as follows; 50 ng *firefly* reporter genes, 15 ng pRL-CMV (*Renilla* luciferase), 50 ng pVgEcR-RXR, pmiR-122 (0 ng, 10 ng, 50 ng, 100 ng, 200 ng, and 400 ng, respectively for Fig. 2B or 100 ng of pmiR-122, 125a and 125b for Fig. 3A) and pcDNA3.1 to obtain a total amount of 515 ng (Fig. 2B) or 415ng (Fig. 3A) of DNA per well. For Fig. 2C, 6 well plates were used for RNA measurement. Amounts of DNA that were transfected were as follows; 150 ng *firefly* reporter genes, 45 ng pRL-CMV (*Renilla* luciferase), 150 ng pVgEcR-RXR, pmiR-122 (0 ng, 30 ng, and 600 ng, respectively) and pcDNA3.1 to obtain a total amount of 1545 ng of DNA per well.

### qPCR Analysis

Total RNA was extracted from NIH3T3 cells with TRIZOL reagent (Invitrogen), followed by DNaseI treatment twice (Ambion), iScript cDNA synthesis kit (Bio-Rad) was used for reverse transcription, and the levels of firefly and *Renilla* luciferase mRNA were examined by MyiQ real-time PCR machine (Bio-Rad) using iQ SYBR Green Supermix (Bio-Rad). The primer sequences were as follows: pGL3-lucF, 5′-CTGATTTTTCTTGCGTCGAGTTT-3′; pGL3-lucR, 5′- GCGCGGAGGAGTTGTGTTT-3′; pRL-lucF, 5′-ACATGGTAACGCGGCCTCTT-3′; and pRL-lucR, 5′-TGCCCATACCAATAAGGTCTGGTA-3′. The amount of the firefly luciferase mRNA was normalized against that of the *Renilla* luciferase mRNA, and the relative mRNA abundance was calculated using Pfaffl Ct method [2].

For ASO-treated mice, RNA was prepared as previously described [22]. Primers used were as follows; NocF, 5′-ACCAGCCAGACATACTGTGC-3′; NocR, 5′- CTTGGGGAAAAACGTGCCT-3′, 45srRNAF, 5′-TGCTGATTGCTTGTTTGGTC-3′; 45srRNAR, 5′-ACACCCGAAATACCGATACG-3′.

### Northern Blot

RNAs were extracted from HEK293 cells by TRIZOL, and equal amounts of total RNA were denatured and fractionated by electrophoresis on a 15% polyacrylamide-8M Urea gel. After electroblotting and cross-linking onto Hybond-N^+^ membrane (Amersham), the blots were probed at 42°C in QuickHyb (Stratagene) with terminally radiolabeled oligonucleotides complementary to miR-122 (5′-ACAAACACCATTGTCACACTCCA-3′), miR-125a (5′-TCACAGGTTAAAGGGTCTCAGGGA-3′) miR-125b (5′-TCACAAGTTAGGGTCTCAGGGA), or U6 (5′-CATCCTTGCGCAGGGGCCATGC-3′), followed by washing with 2× SSC (1× SSC is 0.15 M NaCl plus 0.015 M sodium citrate)-0.1% sodium dodecyl sulfate (SDS), and then with 0.5× SSC-0.1% SDS at 42°C.

### Western Blot

Mouse liver samples were homogenized in RIPA buffer (50mM Tris-HCl, 150mM NaCl, 2mM EDTA, 0.5% Sodium deoxycholate, 1% Igepal CA-630, 5mM DTT), containing protease inhibitor (SIGMA). Equal amounts of each sample were separated by electrophoresis on an SDS-10% polyacrylamide gel before transfer to PVDF membrane (Bio-Rad). The membrane was then blocked with BLOTTO solution (0.1% Tween 20 and 5% dry nonfat milk powder in Tris-buffered saline [TBS], pH 7.4) for 1 hr at room temperature. The membrane was treated with the primary antibody, anti-Nocturnin antibody (Gift of Garbarino-Pico, E.) or anti-TUBULIN (SIGMA) overnight at 4°C. After washing, the blots were treated with secondary antibodies conjugated to horseradish peroxidase, and developed with chemiluminescence Western blotting kit (Roche) or ECL plus (Amersham).
